# Ewald Hering's (1879) “On Muscle Sounds of the Eye”: A translation and commentary

**DOI:** 10.1177/20416695241229019

**Published:** 2024-02-13

**Authors:** Hans Strasburger, Nicholas J. Wade

**Affiliations:** 9183 Ludwig-Maximilians-Universität, München, Germany; Psychology, University of Dundee, Dundee, UK

**Keywords:** eye movements, saccades, afterimages, reading, Hering

## Abstract

Investigations of eye movements were transformed by Ewald Hering in 1879. He developed a novel method for recording them using the muscular sounds attendant on their rapid movements. Brief “clapping” sounds could be heard with the aid of a device like a stethoscope placed on the eyelid and they occurred when afterimages or “floaters” were seen to move. Hering applied the technique to record eye movements during reading and he called the rapid eye movements *Rucke* (jerks in English). Hering published a long review of eye movements and spatial vision later in 1879, but without a description of the muscle sounds. Hering's insightful article has been overlooked and a translation of it into English is presented.

## Introduction

**Figure 1.**
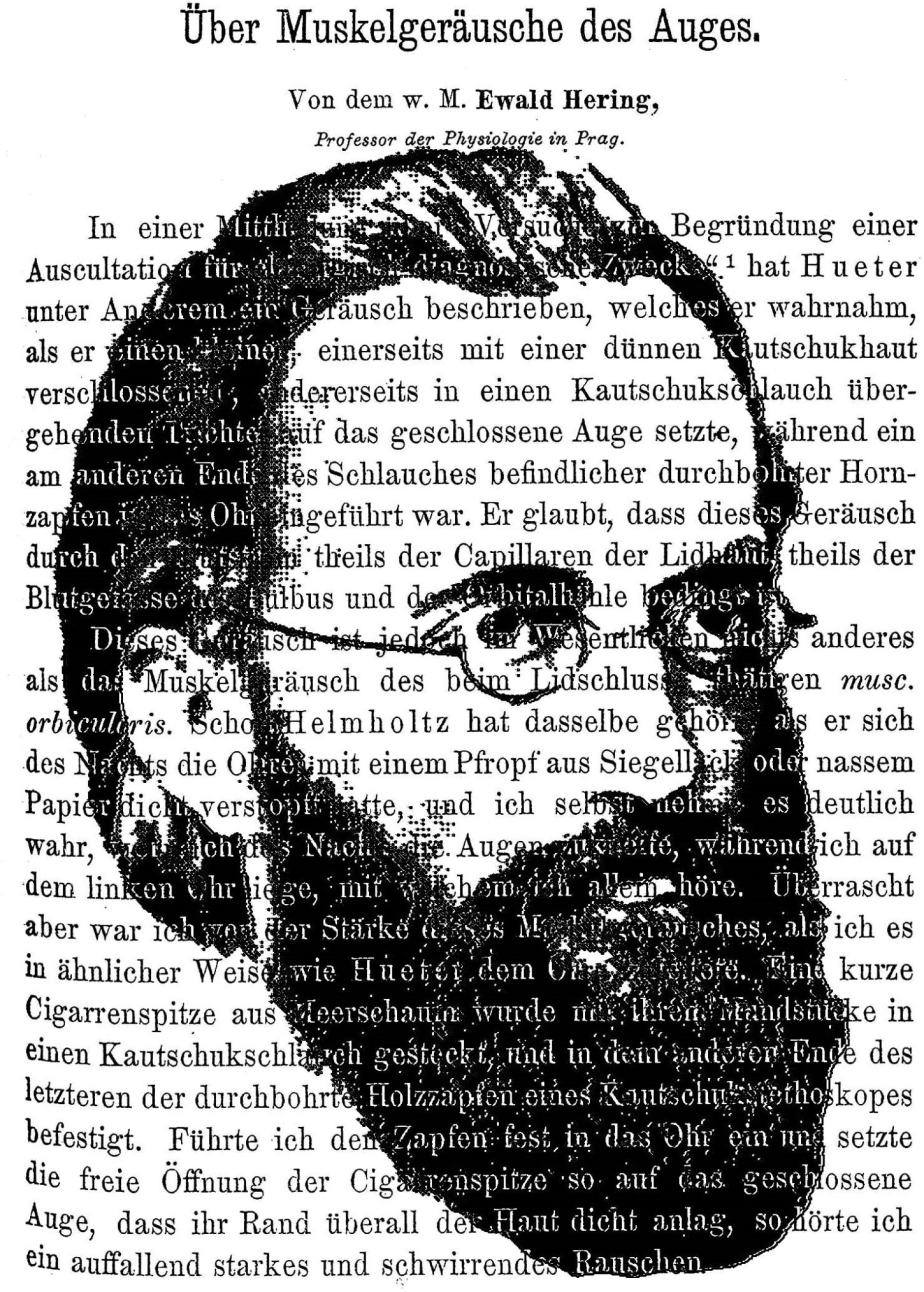
*The sounds of eye movements* by Nicholas Wade. A portrait of Ewald Hering (derived from an illustration in Lesky, 1965) and the first page of his article describing the sounds heard when the eye moves (Hering, 1879a).

Ewald Hering (1834–1918; Figure 1) wrote and experimented extensively on eye movements. He produced a detailed review of the spatial sense and eye movements for Ludimar Hermann's *Handbuch der Physiologie* (1879b) which has been translated into English later by Radde (Hering, 1942). It is likely that the experiments reported in the article translated here (Hering, 1879a) were carried out after the review was completed and before it was published because it is only referred to briefly and obliquely in a footnote (Hering, 1879b, p. 517). Radde did not follow Hering's style of including references in footnotes but placed them at the end of each section. Oddly, the reference to Hering (1879a) is not given in Radde's translation.^1^ Whereas the review was concerned more with the destination and orientation of the eyes following movements, the experiments in the article sought to determine characteristics of the movements themselves using a novel technique. It contains what is probably the first experimental evidence of discontinuous eye movements outside the context of vestibulo-ocular reflexes (see Wade & Tatler, 2005).

Hering's (1879b) review seems to have diverted interest from his brief but insightful experiments published in the same year. His attention was initially drawn to an article by Hueter (1878) in which passing reference was made to the observation that sounds could be heard when a rubber tube was applied to the closed eyelid. Hueter suggested that the sound was generated by blood flow through the capillaries in the lids. Hering (1879a) presented evidence to indicate that the sounds were a consequence of muscle contractions. He attached a rubber tube to a cigar holder and listened to the sounds produced when it was placed on the eyelids and heard a surprisingly strong and whirring roar. When he placed the device on the eye of curarized dogs or rabbits, the sounds ceased even though the blood flow continued.

Having determined that the sounds reflected muscular contractions, Hering distinguished three kinds of sound. Loudest was the whirring sound which he showed to be caused by the orbicular muscle (i.e., the muscle closing the eyelids). By way of relaxing that muscle while applying his technique to the eye lid of an open eye, Hering was able to turn his attention to sounds for the eye movements themselves. Surprisingly, even when he kept his eyes still, he heard a faint, continuous whispering noise (Rauschen), which from today's view might have stemmed from microsaccades. Importantly, however, when moving his eyes in what felt like a continuous manner, this continuous noise was interrupted by regular, “clapping” sounds, reminiscent of the sound of heart beats in auscultation.^2^ Using his experience with afterimages, Hering was able to establish that the sounds were correlated with eye movements. This was further confirmed by movements of floaters, which were accompanied by the clapping sounds.

Hering's study is significant not only because he compared afterimage movements with the sounds of muscular movements, but also because he applied the technique to reading. While reading along a line of text, the clapping sounds occurred regularly. The sounds were louder when the eye moved from the end of one line to the beginning of the next. However, the clapping sounds disappeared when subjects were instructed to fixate a stationary target. It is also of note that he used the term “jerks” (*Rucke* in German) because it is equivalent to using the term “saccades” to describe rapid eye movements if he had been writing in French. Thus, Hering described the discontinuity of eye movements and recognized the class of rotations that we now refer to as saccadic.

Hering was born in Alt-Gersdorf, a small village in the Lusatia (Lausitz) in Saxony. He was educated at the Gymnasium at Zittau and studied medicine at the University of Leipzig, graduating in 1858. He remained in Leipzig for seven more years where he pursued an academic rather than a medical career. His experiments in vision commenced soon after graduating, and he maintained an interest in vision research throughout his life. Hering was appointed professor of physiology at Joseph's Academy (Josephinum), Vienna, in 1865, but his tenure at Vienna was fraught because Ernst Wilhelm Brücke, who occupied the chair in physiology at the university, was a close friend of Helmholtz who was in regular theoretical conflict with Hering (see Turner, 1994). Hering moved to Prague, to succeed Purkinje, in 1870, and finally returned to Leipzig in 1895; he was a colleague of Ernst Mach while at Prague. Despite his teaching commitments, Hering was a very productive and ingenious researcher (see Baumann, 2002). Hering was in the nativist and phenomenological tradition of Kant and Goethe; he contended that each retinal point has local signs for the space values of height, breadth, and depth, and that binocular fusion was physiological. Hering investigated binocular eye movements and argued that the two eyes move as a single unit: his law of equal innervation states that when one eye moves the other moves with equal amplitude and velocity, either in the same or the opposite direction. Eye movements were also implicated in visual direction (see Ono, 1979; Ono & Mapp, 1995).

It is evident that much of the 19th century research on eye movements in Germany was concerned with the geometry of eye position following a movement, rather than the eye movements themselves. The dynamic aspects of eye movements were initially investigated in the context of visual vertigo following body rotation. The method, introduced by Wells (1792), was to compare the apparent motion of an afterimage (formed before rotation) with a real image (viewed afterwards while stationary), thereby demonstrating the fast and slow phases of nystagmus. Both Breuer (1874) and Mach (1875) employed this method. Breuer carried out a series of experiments in which he demonstrated the fast and slow phases of nystagmus and the absence of vision during the rapid return movements. Mach improved on the method by generating an afterimage from observing a flaming magnesium wire: “After stopping, the afterimage moves slowly across the objects clockwise, interrupted by jerklike movements in the opposite direction. The objects then move counterclockwise” (Young et al., 2001, p. 84). The translation of Mach's description uses the term “jerklike” for the rapid returns of the eyes; the German word used was “ruckweise.”

## Translation

**On muscle sounds of the eye.**

From the w. M. **Ewald Hering**,

*Professor of Physiology in Prague.*

In a paper on “Experiments to establish an auscultation for surgical-diagnostic purposes” [“Versuche zur Begründung einer Auscultation für chirurgisch-diagnostische Zwecke”],^a^ Hueter described, among other things, a sound which he perceived when he placed a small funnel, closed on one side by a thin rubber skin and on the other by a rubber tube, on the closed eye, while a pierced horn cone at the other end of the tube was inserted into the ear. He believes that this noise is caused by the blood flow partly from the capillaries of the eyelid skin and partly from the blood vessels of the bulb and the orbital cavity.

This sound, however, is essentially nothing other than the muscular sound of the *musc. orbicularis* which is active in closing the eyelids. Helmholtz already heard it when he plugged his ears tightly at night with a plug of sealing wax or wet paper, and I myself perceive it clearly when I close my eyes at night while lying on my left ear, with which I hear alone. But I was surprised by the strength of this muscular sound when I directed it to the ear in a similar way to Hueter. The mouthpiece of a short cigar made of meerschaum^3^ was inserted into a rubber tube, and the pierced wooden spigot of a rubber stethoscope was attached to the other end of the tube. When I inserted the spigot firmly into the ear and placed the free opening of the cigar tip on the closed eye in such a way that its edge was close to the skin everywhere, I heard a strikingly strong buzzing noise.

I have also observed this noise in dogs and rabbits and it disappeared completely by curarization of the animal. Since the circulation in the eye is not abolished and the blood pressure is almost unchanged, it follows that the noise is not a vascular noise.

Further preliminary observations then showed me that the sounds of the muscles of the eyeball can also be heard in this way, and so Hueter's statement became the starting point for the present small investigation. But before I go into the latter itself, I would like to make a few remarks about the “capillary sound of the fingertips” described by Hueter, which gave him the first impulse for his experiments, and which I examined more closely years ago. Collongues^b^ once based his system of dynamoscopy on this long-known sound, and it has now become the fundamental observation for Hueter's dermatophony. But Brown-Séquard, as I see from a remark by van Hasselt,^c^ was right in declaring it to be a muscular sound.

A stick made of wood or metal, which ends in a suitably shaped button inserted into the ear, sends a clear muscular sound to the ear as soon as it is held firmly with the fingers, while the sound is only faintly audible when it is touched softly. By holding the stick with the hand, various muscles are engaged and the vibrations of these are transmitted to the ear through the fingers and the stick. The sound becomes very strong when muscles that act on the bones of the fingers involved are put into powerful action.

Instead of using any kind of feed-line device, one can also use the naked finger, the tip of which is inserted into the external auditory canal. As long as the finger remains completely still in the auditory canal, one hears, as is well known, a faint murmur (bourdonnement), which is interrupted at every slight movement of the hand, as it easily occurs involuntarily, by short cracking or creaking noises (pétillements, grésillements according to Collongues), which have nothing to do with the muscular noise. However, I must conclude that the evenly sustained murmur is a muscular noise from the fact that it disappears when I try to avoid any active tension in the muscles of the upper extremity. When I lie down on my left side at night after inserting the index finger of my left hand into my left ear, I can make the noise disappear completely by positioning my head, arm and hand appropriately, whereby all the muscles of these parts are completely passive, yet the circulation is not inhibited. I still have weak subjective auditory sensations, but they are of a completely different kind and are exactly the same as those I notice in the silence of the night without inserting my finger.

If I now tense any muscle of the hand after the muscle noise has been completely eliminated in the manner mentioned, I immediately hear a muscle noise again. Even if I have inserted the finger while sitting or standing, so that the arm is held in position by muscular action, I can immediately change the weak hissing noise that I hear into a much stronger and more buzzing noise if I innervate individual muscles of the hand, for example, strongly adduct the thumb.

I have also convinced myself in another way that the blood movement in my fingertip does not make a noticeable contribution to the noise, which, by the way, I had not expected anyway. Namely, if I make the skin of the fingertip as bloodless as possible by inserting a tight rubber ring from the fingertip to the second phalanx and then insert it into the ear, the muscle noise remains unchanged. After all, a small difference could have resulted from the fact that the bloodless and therefore collapsed fingertip does not close the auditory canal as well as the blood-rich fingertip if not inserted deeper than the latter. However, I could not find such a difference.

### 1. Description of the Apparatus Used for the Examination of Eye Muscle Sounds and the Sources of Error to be Observed in its Use

I had three small conical tubes made of hard rubber, modeled on the above-mentioned cigar tip, which were 15, 10, and 5 mm at the wider end, all 3 mm in diameter at the narrower end and 5, 4, and 3 cm in length, respectively. In the following, I will refer to them as sound funnels. The narrower end was inserted into the 42 cm long rubber tube of 5 mm in total diameter and 3 mm in clearing diameter, which carried the polished and pierced wooden peg mentioned above at the other end. The largest of the three funnels proved to be wide enough to transmit the muscle sound from any skeletal muscle to the ear with sufficient clarity. For listening to the muscles of the face, the eyes, the lips, the tongue, even the smallest one proved to be sufficient. Yet I found the medium-sized one to be the most suitable for the investigation of eye muscle sounds. Preliminary control experiments with these small listening devices gave me the following results:

When I placed the peg in the ear and held its protruding end that merged with the tube with my fingers, I heard a very clear muscular sound, which, as follows from the above, was supplied by the fingers. This initially led to the requirement not to hold the cone with the fingers, but to fit it into the ear in a way that it stuck by itself.

After this was done, I placed the funnel at the other end of the tube quietly on the table. Even now, I still heard a faint muffled noise, which became higher and fainter when I closed the opening of the bell with a small rubber plug or otherwise. The cause of this noise was finally found to be the contraction of my masticatory muscles; without thinking about it, I had pressed my jaws weakly against each other. When I lowered my lower jaw a little, the noise disappeared.

But soon afterwards I heard it again quite clearly, and I found that my head position was the cause, because the noise increased considerably when I tilted my head back or to the side. When I lowered my head slightly forward, it disappeared completely. Apparently the noise was caused by the tension of the *sternocleidomastoid muscle.*

When I had thus found out the correct position of the head and the lower jaw, the noise was constantly absent. I should note that, apart from right-sided deafness, my hearing in the left ear is not better than most people's, but perhaps a little worse, and that it is therefore easily possible for others to hear yet other sounds coming from more distant muscles under the conditions I have just described. I do not mean the sounds of the deliberately strongly contracted muscles of the face, tongue, palate or throat, for I also hear these very clearly when the cone is in the ear, but sounds which are caused either by an unconscious weak activity of the above-mentioned or yet other muscles.

All the muscular noises mentioned here, which are transmitted directly to the auditory organ through the bones of the skull, I sometimes do not hear at all when the ear is open, sometimes extremely faintly when everything is completely still, yet clearly when I block the ear like Helmholtz. The insertion of the described apparatus into the ear thus has a similar effect as the plugging of the ear. If, however, I insert the pierced cone of the apparatus alone, that is, after disconnecting the tube, the noises are not heard, whereas they are heard when the tube is still connected to the cone but the funnel is removed. This is therefore a resonance in which the tube plays the main role. This is also evident from the fact that the noise immediately changes in height and intensity when the tube or the funnel connected to it is closed at the free end.

If the tube or the funnel is closed after the cone has been inserted into the ear, the air in the tube can be compressed in case the cone happens to close the auditory canal airtight, thus changing the tension of the eardrum and thereby introducing a new complication. This can also happen if the funnel is placed tightly on the skin in order to auscultate the muscles underneath. I therefore had a small side opening of 1 mm diameter made on the funnel, which is closed by a springy and leathered flap as soon as the funnel is put on. If the peg does not close the ear canal airtight, such precautions are not required. However, since the muscle noises transmitted through the funnel and tube are heard more clearly the better the peg fits the auditory canal, the case of airtight closure and compression of the enclosed air column may well occur.

When I gently grasped the funnel with my fingers and brought its opening into contact with the surface of water in a small beaker, with the air in the bell closed off by the water, I again heard a strong noise. That noise was caused by the fact that I held the funnel with my hand; for the tighter I grasped it, the louder the noise became, and when I fastened the immersed funnel to a clamp instead of holding it myself, the noise disappeared, but immediately returned when I grasped the glass or the clamp firmly somewhere; and indeed the noise became stronger the tighter I grasped. So once again it was a muscular noise transmitted through the fingers.

Now I closed the funnel with a thin rubber membrane. As soon as I touched the latter with a fingertip while the funnel was fixed in the clamp, I heard the muscle noise; likewise when I took the unattached funnel between my fingers and pressed hard without touching the membrane. If, on the other hand, I touched it quite gently, I heard nothing. But if I brought the membrane of the softly grasped funnel into contact with any strongly convex surface, the noise appeared again. In order to have a moist surface and thus to avoid the creaking noises which are caused by the friction of the rubber membrane against wood, metal or the like, I used in particular a freshly cut eyeball from a dog. This eyeball, when touched by the rubber membrane, made a very pure sound, which resembled a muscle noise. So I had to conclude again that only holding the funnel with my fingers caused the noise. For while I have a sure hand and am able to prepare under the microscope with an inverted image, even at higher magnifications, I cannot avoid the invisible vibrations that are caused by the activity of my muscles. So I brought the membrane of the funnel back into contact with the eyeball, but fixed the funnel with the clamp and now heard no more noise. But when I took hold of the eyeball or the clamp with my fingers, the noise returned.

I must therefore find Hueter's use of a membrane to close the funnel objectionable. He probably only used it in order to be able to place his funnel, which seems to have had a much wider opening than mine, on areas of the skin where, because of the shape of its surface, the funnel's rim could not seal airtight, so that the membrane was to take over the closure. **Such a funnel, however, when held by hand, makes a muscular noise audible on every convex surface, as soon as the rim of the funnel does not sit firmly on the surface all around and the membrane does not nestle completely to the latter. In this case, however, the membrane is useless.**^4^

### 2. Description of the Muscle Sounds Perceived at the Eye

When I place the funnel's free opening on the closed eye, I hear, as I said, a very strong muscular sound which, because of its intensity, cannot be confused with the above-described muscular sounds supplied by the bones of the skull. I hear the sound strongest with the largest, weakest with the smallest funnel. Yet I found the medium sized one the most suitable, which can easily be placed on the eye to form an airtight seal. The sound can be heard from both the upper and lower eyelid. I found no difference in its strength when I placed the funnel alternately on the left or right eye, while the peg was always in the left ear. When I place the funnel on the forehead for control while grasping it very lightly, I do not hear the noise; but here too a weaker muscular noise appears when I furrow my brow or bring the *m. corrugator supercilii* into contraction.

When, while both eyes are closed and the funnel is on one of the upper lids, I try to slowly open both eyes, the examined eye's upper lid is held by the funnel and only the other eye opens. At the same time, however, the sound in the now passively closed eye changes immediately; in particular, it becomes noticeably fainter, as if it were coming from a greater distance. The analogous change in the sound occurs when I place the funnel on the lower lid of the closed eye and then open the eye. This sound, which is audible even when the orbicular muscle is as relaxed as possible, was the main subject of my investigation and must be strictly separated from the sound of the active *m. orbicularis*. The great difference between the two sounds became very apparent to me when I first placed the funnel on the lower lid of the open left eye and then closed only the right eye. As long as both eyes are open, the orbicular muscles are slack and I therefore only hear the weaker sound. But when I close the right eye, I involuntarily innervate the left *m. orbicularis* as well, because I am unable to fully loosen the association of the muscles on both sides, and although the left eye remains open, its palpebral fissure becomes noticeably narrower. The unintentional associated activity of the left sphincter that takes place immediately now causes the stronger orbicular sound, as I will briefly call it.

The difference between the two sounds, apart from the greater strength of the orbicular sound, lies in the fact that the latter is at the same time strongly whirring or vibrating [stark schwirrend oder vibrirend], while the other sound is more like a uniform rustling [Rauschen], apparently coming from a greater distance. You can hear it no matter where you place the sound funnel on your eye with the *m. orbicularis* as relaxed as possible. Since my eyeballs protrude sufficiently beyond the orbital rims, I can place the medium-sized funnel on the lower or upper lid at various points with the eye open. Finally, I can also place the smallest funnel directly on the sclera. With all these different ways of applying the funnel, I hear the sound. Since the latter, like the orbicular noise, cannot be eliminated at will, but is always heard, and in order to distinguish it from this noise as well as from other noises to be described shortly, which are only of momentary duration, I will call it the continuous noise.

Namely, while observing it one hears very short, muffled-clapping noises [dumpf klappend] that follow each other at irregular intervals. At first, they are easily overlooked because one's attention is too exclusively focused on the continuous noise. These momentary sounds, as I will call them, are best compared to heart sounds. If one were to auscultate a heart beating quite irregularly at some distance from the heart area, one would obtain very similar sound sensations.

The momentary noises are demonstrably the result of unintentional, jerky movements of the eyeball. While paying attention to the continuous noise, one is not aware of how restless the eyes often are, and especially not of the fact that their movements are jerky. If one fixates one point firmly, the momentary noises disappear, only to reappear as soon as movements of the eyeball occur again as a result of fatigue or temporary inattention.

Those who are not very experienced in fixation do well to create a long-lasting after-image, for example, of a small white disc on a black background, and only then to fixate a marked point on a monochromatic background: they will then convince themselves that every momentary noise of the eye corresponds to a shift in the after-image. Likewise, those *mouches volantes,* which are set into slight real (not merely apparent) motion by eye movements, offer a convenient control of the aforementioned unintentional fluctuations of the gaze.

The momentary sounds can be observed very well when reading. While the gaze seems to glide steadily along the line, the momentary sounds betray the jerky movement of the eyeball. If the gaze jumps from the end of one line to the beginning of the next, one hears a particularly clear sound, which, however, is somewhat longer and, in contrast to the short tapping that one usually hears, has something scraping or rubbing [etwas Schabendes oder Reibendes] about it; it relates to the other instantaneous sounds in much the same way as a heart murmur to a heart sound.

While in my opinion there can be no doubt about the cause of the orbicular noise and the momentary noise, the continuous noise causes greater difficulties for an explanation.

### 3. About the Cause of the Muscle Noise that is Always Audible in the Open Eye

That the continuous noise is also a muscular noise, I must conclude from the fact that, firstly, it has the character of such a noise, secondly, that it is generally the stronger the more the eye is deflected from its central position, and thirdly, that I do not hear the noise in the eyes of curarized animals, although the blood movement in the eyeball and in the orbita continues vigorously.

First of all, I tried to find a gaze position in which the continuous noise would completely disappear. I assumed that there could be an eye position in which the innervation of all muscles of the bulb was minimal or equal to zero. However, I did not succeed in finding an eye position in which the noise would have become even indistinct. It was weakest when the visual plane was moderately lowered with weak symmetrical convergence of the lines of sight. On the other hand, I easily succeeded in amplifying the sound and at the same time making it rougher and somewhat buzzing, if I kept the eyeball considerably deflected from the middle position, no matter in which direction.

Accordingly, if the continuous noise were to emanate only from the muscles of the eyeball and not also from those of the eyelids, it would have to be concluded that there is no gaze position in which either individual or perhaps all eye muscles are not moderately innervated. Though this assumption would not correspond to the prevailing views, it would not be paradoxical. For it might be thought that the eye muscles, like certain mimic muscles, are under tonic innervation, especially when the eyes are open and the attention is turned to the impressions of the sense of sight.

Before one would be forced to make such an assumption, however, it would have to be proven that the continuous *murmur* is neither caused by the m*. levator palp. sup.,* nor also by those muscle fiber tracts which are usually grouped together as the *m. orbicularis.* The main question is whether the *m. levator* is always active as long as the palpebral fissure is open and whether a slight closure of the palpebral fissure is possible by the mere slackening of this muscle or only with the help of the *m. orbicularis*.

Dogs, cats, and rabbits do not have their eyes closed after death, as do humans as far as my experience goes. A moderate opening of the eye would therefore correspond to the equilibrium of the elastic forces of all the parts under consideration here. Nevertheless, it still needs to be investigated in more detail what part the tonic activity of the *m. orbicularis* plays in the closing of the eyes during sleep. I carefully placed the aforementioned funnel on the eye of a sleeping boy and indeed heard a faint muscular noise, although no visible reflex movement of the eyelids occurred as a result of the contact. In the case of complete paralysis of the orbicular muscle, closure of the eyelid is impossible and during sleep the palpebral fissure is moderately open. “The upper lid of the eye,” as v. Stellwag^e^ says, “lies slack against the bulb, while the lower lid usually protrudes from the eye or is even turned inside out.” If this extreme slackening of the orbicular muscle is already apparent in very recent cases and is not only the result of secondary changes, of which I have no knowledge, then one would even have to assume that there is a tonic innervation of this muscle not only during sleep but also when awake. If I tilt the visual plane downwards as far as possible, my upper eyelid descends almost to the end of the palpebral fissure, while the edge of the lower eyelid descends only very slightly. I do not hear the characteristic orbicular noise mentioned above, but only the continuous noise. Nevertheless, I do not want to assume that the strong lowering of the upper eyelid is only due to the slackening of the *m. levator.* Rather, individual fibers of the orbicular muscle acting on the upper lid are probably involved. The accuracy with which the upper lid follows every downward or upward turn of the eyeball in such a way that a segment of the iris of almost the same size is always covered by the lid is striking, while the lower lid margin changes its position so little that its slight displacement can easily be regarded as a purely passive displacement caused by the displacement of the conjunctival fold.

In certain cases of *ptosis paralytica,* it would perhaps be possible to deduce, from the behavior of the upper eyelid, which position of the latter corresponds to the complete slackening of the *m. levator* at various inclinations of the plane of vision. The examination of the muscle sounds in the various forms of paralysis of the eyelid and eye muscles would also provide some interesting information if it were carried out by someone who has already studied the muscle sounds of the healthy eye more closely.

According to what has been said, it must remain undecided for the time being whether there is an inclination of the visual plane at which both the *m. levator* and the *m. orbicularis* are completely inactive, and to what extent, at different inclinations of the visual plane, the continuous noise is partly caused by the activity of these muscles.

But if, on the one hand, there were a position of the plane of sight in which the eyelid muscles were completely passive and, on the other hand, a position of the plane of sight in which all the muscles of the bulb were inactive, then the further requirement would also have to be fulfilled that this position of gaze corresponded precisely to that inclination of the plane of sight if all the muscles in question here were otherwise inactive. Only in this case could the continuous noise disappear completely.

### 4. Study of the Innervation Laws of the Eye With the Aid of Muscle Sounds

As I have shown in detail elsewhere,^f^ with one and the same position of the line of sight of one eye, the motor innervation of same eye and accordingly also the activity of its musculature is very different, depending on the simultaneous position of the line of sight of the other eye. Depending on whether the fixated point on an unchanged line of sight is moved into the distance or into the near, the activity of the muscles acting on the bulb, apart from the accommodation, also changes. Whether the other eye is covered or the point in question is fixated binocularly is essentially irrelevant.

This difference in innervation and muscular action is revealed firstly by a change in the orientation of the retina, that is, by a different inclination of the vertical or horizontal dividing lines to the plane of sight, and, as I have shown, this inclination may differ by up to 5° or 6° in near vision from that in far vision.

Secondly, an essential difference in innervation can be theoretically deduced from the general law of binocular innervation that I have established, for the correctness of which I believe I have provided sufficient evidence elsewhere. According to this law, both eyes are generally innervated simultaneously and equally during vision, and this also applies to the asymmetrical convergence positions of the eyes, in which one should assume, from the outset, a different innervation of the eyes. Let us imagine, for example, that we fixate on a point lying straight in front of us at a great distance, with the lines of sight horizontal and parallel; in front of the left eye, on its line of sight and about 10 cm away from the eye, there is a needle point which initially would give very blurred double images. In order to see this needle singly and clearly, the eyes would have to change from their parallel position to an asymmetrical convergence position, whereby the left eye would not have to change its position at all, but only the right eye would have to turn strongly inwards. From the above-mentioned law of innervation it follows, however, that the left eye also experiences strong innervation, but that because the *m. rectus externus* and *internus are* innervated simultaneously, and their antagonistic traction forces cancel each other out, this innervation does not result in a changed position of the line of sight.

The analogy applies to all cases in which, with the position of one line of sight unchanged, the gaze passes from a distant to a near point not situated in the median plane of the head. Only if the near point of fixation is situated in the median plane itself does this law find an exception. If one fixates a median point binocularly and then directs one's gaze to a distant point which is on the line of sight, for example, of the left eye, then according to the law of innervation the central quality of the innervation changes insofar as the innervation for the common right turn of both eyes takes the place of the convergence innervation, but the tension of the, substantially involved, *m. rect. internus* could remain unchanged; only it would no longer be associated with the *m. rectus internus* in its activity, but with the *m. rectus externus of* the right eye.

In such special cases, therefore, no change in the muscular action follows from the law of innervation for that eye on whose unmoved line of sight the point of fixation moves from near to far or vice versa. But here, too, at least the inclination of the dividing lines to the visual plane changes and proves to us that the activity of the eye muscles undergoes a change in spite of the unchanging position of the line of sight, which should essentially affect the oblique muscles.

In general, it thus follows that whenever, with an unchanged position of the line of sight of an eye, the binocular fixation point shifts along that line of sight, an altered action of the muscles of this eye results.

Now, does this difference in muscle action also manifest itself in a difference in muscle sound? This is indeed the case, and in a very striking way. If, for example, I place the bell on the lower lid of the left eye, while I first fixate on a distant point lying straight in front of me, and then turn my gaze towards a needle point lying close to me on the left line of sight, whereby the left eye does not change its direction, while the right eye is turned inwards, I hear the continuous noise of the left eye clearly increase and, when the needle is very close, even take on that more whirring character, as it also shows itself in particular in an eye that is turned strongly outwards or inwards. This stronger or vibrating muscle noise lasts as long as I fixate the needle, but immediately changes back to the weaker continuous noise when I look again at the distant point. That this is not a muscle noise coming from the right eye emerges from the fact that the noise cannot be heard when I place the funnel on my forehead near the root of my nose.

The change in the sound during close-up vision is so clear that even a second person notices it immediately. I have had several experienced observers (Prof. Knoll, Prof. Sigmund Mayer, Dr. Biedermann) auscultate while I was doing the experiment, and they were all able to tell immediately just from the change in the sound whether my eye, which remained in the same direction, was accommodated for near or far.

Whether the other eye is open or covered with the hand during the experiment makes no difference, because in both cases the innervation of the double eye is the same.

Less noticeable, but still quite distinctly, is the amplification of the sound during near vision when the needle tip is located in the median plane.

Thus it can also be proven from the muscular sounds that, with the same position of the line of sight, the eye muscles are active in different ways, depending on whether the fixated point is near or far.

To start with, the amplification of the continuous noise during near vision suggests a correspondingly amplified muscular action, as is indeed required by the Innervation Law mentioned, if the needle tip is not in the median plane. However, in the case of strong convergences, another circumstance may come into consideration. Such convergences are unfamiliar to us, exhausting, and accompanied by an uncomfortable feeling in the eye area. Unaccustomed movements and positions of our limbs, however, are insecure and less steady than those we are used to, because we are not trained in the innervation required thereby. The discontinuity of the innervation leads to discontinuities in the muscle action and thus perhaps to stronger and especially more clearly vibrating muscle noises.

I already have the feeling of exertion at degrees of symmetrical convergence that only require such an inward turn of the eye as takes place without any uncomfortable feeling when only one eye is turned inwards and the other outwards. So it is not the inward turn of the eye as such that strains me and causes an unpleasant feeling, but the simultaneous inward turn of both eyes. The cause of this feeling of exertion can be peripheral or central. If it is peripheral, it must be sought either in the simultaneous accommodation tension or in the fact that when the eye is turned inwards for the purpose of convergence, greater muscular forces come into action than when the eye is turned inwards to the same extent for the purpose of fixing a distant object at the side. The law of innervation I have established offers no reason to assume increased muscular action in the case of symmetrical convergence. Nevertheless, such an action could also be imagined here, quite apart from a change in the activity of a *m. obliquus,* which is not improbable because of the changed position of the dividing lines. Firstly, since this is a strong innervation that is seldom practised, it could irradiate muscles whose activity is not at all necessary to produce convergence, as we also see unintentional co-movements occurring in unpractised and strenuous movements; or, in case it were true that every direction of our will towards perception by the sense of sight brings about a tonic innervation of all the eye muscles, then this tonus could become all the stronger the greater the difficulties that stand in the way of the desired clear perception. In both cases, the muscles of the eye would not only be more active in general, but the *m. rect. internus in* particular could experience greater tension.

What has been discussed here for strong symmetrical convergences also applies to strong asymmetrical ones.

I did not wish anything left unmentioned that, as far as I can see, could be taken into consideration here, but I believe that for the non-strenuous degrees of convergence the explanation given above from the law of innervation, or from the altered orientation of the eye at near vision, is sufficient.

Based on the consideration that a simultaneous stronger tension of the *m. rect. externus* and *internus* must cause a noticeable increase in the intraocular pressure, I had my retinal vessels observed some time ago in the case of strong asymmetrical convergence of the lines of sight, after I had previously instilled atropine. Dr. Becker, now a professor in Heidelberg, was kind enough to take over the examination. But although he found the symptoms of increased pressure in near vision sometimes indicated, yet not definite enough to be able to draw a firm conclusion. I did not use a tonometer.

At the time, the reliable proof of an increase in intraocular pressure would have been valuable to me because I thought of the possibility that the central innervated processes, which correspond to the tension of the *rectus internus* on the one hand and the *rectus externus* on the other, were in such an antagonistic relationship to each other that they could cancel each other out. In this case, the law of innervation I have posited could be correct, without the eye positions mentioned having to result in the simultaneous activity of the two antagonistic muscles. For if the antagonistic processes of innervation already cancelled each other out in the central organ, there would be no innervation of the muscles themselves. The amplification of the muscle noise also speaks against this, which makes a really intensified muscle action and thus at the same time an increase of the intraocular pressure probable.

### Notes to the Original Paper

^a^Centralblatt für die medicinischen Wissenschaften, 1878, No. 51.

^b^Gazette médicale de Paris, 1856, 1858, 1859, 1860 and 1862.

^c^Die Lehre vom Tode und Scheintode, S. 58 [The Doctrine of Death and Apparent Death, p. 58.]

^d^Sitzb. d. mathem.-naturw. Cl. LXXIX. Bd. III. Abth. [Sitzungsberichte der mathematisch-naturwissenschaftlichen Classe, Band 79, Dritte Abtheilung. (The note appears on the bottom of page 145; it is not referenced in the text).]

^e^Lehrbuch der Augenheilkunde, III. Auflage, S. 831 [Textbook of Ophthalmology, III. edition, p. 831].

^f^Die Lehre vom binocularen Sehen, p. 9 [The Doctrine of Binocular Vision, p. 9].

## Commentary

Speculations about how the eyes move have long been entertained. For example, Porterfield (1737) referred to “the quick and almost continual Motion of the Eye, whereby it is successively directed towards all the Parts of the Object in an Instant of Time” (Porterfield, 1737, p. 186). However, such speculations were not based on observation but on theory. It was known that visual acuity was highest around the fovea and declined peripherally which did not accord with visual experience. Despite proposing that the eye moved rapidly, Porterfield (1759) denied that such occurred in post-rotary vertigo. Instead, he stated that the eyes did not move because he was not aware of them moving! It was only when there was an index of eye movements that they could be investigated experimentally. This index was the comparison between an afterimage and a real image—a fact appreciated by Wells (1792) and applied to eye movements following body rotation. That is, an afterimage is stabilized with respect to the eye whereas an external image is not; relative movement between them indicates movement of the eye. The technique enabled Hering to confirm that the clapping sounds he could hear in his stethoscopic device when reading reflected movements of the eye.

Hering made some general statements about how the eyes move in his *Handbuch* chapter, but not with the detail given in the article:

In ordinary seeing the visual point constantly changes its position; for, in order to recognize the outer objects, we must examine one after another, all their separate parts and endeavor to perceive them with utmost acuity. This can be accomplished only by bringing their images upon the two retinal centers. With small objects the mere turning of the eyeballs in their sockets suffices. But if the object of regard is of large dimensions, we resort to turning the head, and if necessary, the upper body, and finally even changing our location. Rarely do we devote our attention to one spot, even for the duration of a second. Rather does our glance spring from point to point, and in its wanderings it is followed slavishly by the visual point, in whose movements we have greater agility than in any other movement, for no organ is so continuously used as the visual organ. (Hering, 1942, p. 83)

Hering's (1879b) observations above are little more than an elaboration of Porterfield's (1737) speculations whereas his experiments in the article translated here placed the investigation of eye movements in activities, like reading, on an experimental footing. Moreover, in addition to determining that the sounds reflected contractions of the ocular muscles, Hering relates the quieter almost continuous background “noise” to small involuntary movements of the eyes. Ocular instability with attempts to maintain fixation had been noted by Darwin (1786): when trying to form an afterimage of a circular piece of red silk, green crescents were briefly seen around the circumference. Hering provides probably the first physiological evidence of such ocular instability. Moreover, he tried to find the eye position in which sounds were at their minimum.

Much of Hering's discussion of eye movements relates to their binocularity and this is an aspect the article shares with the chapter. What has become known as Hering's law of equal innervation stated that: “The two eyes are so related to one another that one cannot be moved independently of the other: rather the musculature of both eyes reacts simultaneously to one and the same impulse of will” (Hering, 1868, 1977, p. 17). He is able to provide support for his binocular law with the novel sound technique for recording muscular activity in the eyes: “Whether the other eye is open or covered with the hand during the experiment makes no difference, because in both cases the innervation of the double eye is the same.” Hering uses the term “double eye” to denote what is now referred to as “the cyclopean eye” (see Wade, 2021).

Hering's research using this novel technique has been neglected because it has been overshadowed by Javal's articles on eye movements published at around the same time (see Wade & Tatler, 2005, 2009; Wade et al., 2003). Javal (1879) gave a brief description of “saccades” during reading and he is generally considered to have instigated research in eye movements during reading. In fact, however, Javal said virtually nothing about eye movements in his eight essays on the physiology of reading, published in 1878 and 1879. Saccades were only mentioned briefly on the penultimate page of his final article. Measurements of eye movements during reading were carried out in Javal's laboratory by Lamare and reported in passing by Javal: “Following the research of M Lamare in our laboratory, the eye makes several saccades during the passage over each line, about one for every 15–18 letters of text. It is probable that in myopes the eye reacts with a rapid change in accommodation with every one of these saccades” (Javal, 1879, p. 252). It was Lamare who observed and recorded the jerky or saccadic movements during reading in 1879. However, he did not describe his experiments until 13 years later. He tried various methods, including observing the eyes of another person, but:

The method that gives the best results is one by which the movements are heard via a drum with an ebonite membrane in the centre and to which a small tube is attached; the tube is in contact with the conjunctiva or eyelid and is connected to both ears by rubber tubes … The apparatus yields distinctive sounds which an assistant can count and add, and note for each line. The return movement of the eyes to the beginning of a line gives a longer and louder noise that is easy to recognise; one counts the number of *saccades* from the start of the line to be able to *note* the number of divisions that occur in a line (Lamare, 1892, p. 357).

Javal (1879) presents Lamare's results but not the method he applied in obtaining them. These were described by Lamare (1892) but he does not provide any indication of prior work on the muscular sounds nor the validation of the sounds with afterimage movement. The technique he applies also bears a striking similarity to that developed by Hering, and it is hard to avoid the conclusion that Javal and Lamare drew upon Hering's (1879a) publication. Javal was familiar with the German literature as he had jointly translated Helmholtz's *Handbuch* into French (Helmholtz, 1867).

Javal's term “saccades” is now generally accepted rather than Hering's “Rucke,” Brown's (1895) “jerks,” or Dodge's (1903) “type I” eye movements. It was Dodge (1916) who suggested that “saccades” should be adopted: “German and Scandinavian writers are commonly using the descriptive class term ‘saccadic’ to denote the rapid eye movements for which we have only the arbitrary name of ‘type I.’ I am not sure with whom the term originated, but it seems worth adopting” (1916, pp. 422–423). More details of this history can be found in Wade et al. (2003) and Wade and Tatler (2005, 2009).

The relatively short article translated here (Hering, 1879a) can be considered to have had greater significance for eye movement research than the long review chapter (Hering, 1879b) that followed it. The application of a novel technique for recording how the eyes move opened up a research area that continues to expand. Hering is deservedly considered as a pioneer of eye movement research (Wade, 2010).

As a final point, the article is a delightful example of Hering's rigour and ingenuity in pursuing a question that proves open to experimental examination. He poses a succession of problems that he proceeds to solve before satisfying himself that the sounds derive from muscle activity of the eyes. He then applies the method to answer old questions or to confirm his own speculations. It reads rather like a Jamesian “stream of consciousness” account of the scientific enterprise in action.

## Notes

1. Hering's paper is somewhat difficult to come by even today: The “mathematisch-naturwissenschaftliche Classe,” in whose reports Hering's paper is published, was at the time divided into three sections (“Abtheilungen”, abbreviated “Abth.”). Hering's paper is in Section 3 (abbreviated “III. Abth.”), covering physiology, anatomy and theoretical medicine; that third section was added only a few years earlier, in 1872. However, the essential section specifier is all-too-often omitted in standard references, making it hard or impossible to find the paper. Moreover, the fact of the class division is not even apparent in many libraries and repositories as of this writing, such that Section 3 is then missing altogether. Note also that, adding to the confusion, the section number is given in Roman numerals and is located *before* the “Abth.”, not after, as would be standard in an English reference. So, “LXXIX. Bd. III. Abth.” translates to “Vol 79, Section 3.” The article is reprinted in volume 1 of Hering's (1931) *Wissenschaftliche Abhandlungen*.

2. *Auscultation* (from Latin “to listen”) is the technique for examining the respiratory or circulatory system using a stethoscope. The technique is used here in a surprising, unexpected context.

3. *Meerschaum*, or Sepiolite, is a soft white lightweight mineral, often used for making tobacco pipes. To German readers it is well known through Wilhelm Busch's famous 1865 cartoon novel “Max and Moritz,” where, in Chapter 4, Lehrer Lämpel's *meerschaum pipe* is famously, and dramatically, made to explode.

4. In the original, emphasis in that sentence is by typesetting with spaced letters, not bold font. Spaced-letters formatting is also used for emphasizing headlines and author names throughout.
